# Plasticity of bone marrow-derived cell differentiation depending on microenvironments in the skin

**DOI:** 10.3389/fphys.2024.1391640

**Published:** 2024-04-18

**Authors:** Junko Okano, Takahiko Nakagawa, Hideto Kojima

**Affiliations:** ^1^ Department of Plastic and Reconstructive Surgery, Shiga University of Medical Science, Otsu, Japan; ^2^ Department of Regenerative Medicine Development, Shiga University of Medical Science, Otsu, Japan; ^3^ Department of Biocommunication Development, Shiga University of Medical Science, Otsu, Japan

**Keywords:** bone marrow-derived cell, wound healing, scaffold, regeneration, skin, skin regeneration, vasculogenesis, lymphangiogeneis

## Abstract

Bone marrow-derived cells (BMDCs) are heterogeneous populations in which not only pluripotent stem cells, namely, hematopoietic stem cells (HSCs), mesenchymal stem cells (MSC) but also endothelial progenitor cells (EPC) are involved. BMDCs contribute to the maintenance of homeostasis and recovery from disrupted homeostasis as the immune, endocrine, and nervous systems. The skin is the largest organ in which various tissues, such as the epidermis, dermis, skin appendages (i.e., hair follicles), fats, muscles, and vessels, are tightly and systematically packed. It functions as a physical barrier to block the invasion of harmful substances and pathogenic microorganisms and properly regulate water evaporation. The skin is exposed to injuries from external stimuli because it is the outermost layer and owing to its specificity. Recovery from physical injuries and DNA mutations occurs constantly in the skin, but medical treatments are required for impaired wound healing. Recently, conservative treatments utilizing scaffolds have attracted attention as alternatives to surgical therapy, which is highly invasive. Against this background, numerous scaffolds are available in a clinical setting, although they have not surpassed surgery because of their distinct disadvantages. Here, we discuss the plasticity of BMDCs in the skin to maintain homeostasis, in addition to their critical roles on recovery from disrupted homeostasis. We also share our perspective on how scaffolds can be developed to establish scaffolds beyond surgery to regenerate skin structure during wound healing by maximally utilizing the plasticity of BMDCs.

## Introduction

BMDCs include hematopoietic stem cells (HSCs), mesenchymal stem cells (MSC), and endothelial progenitor cells (EPC), all of which are typically located in the bone marrow (Golle et al., 2017). Occasionally, they circulate in the peripheral blood, but the precise mechanism remains unknown (Budkowska et al., 2018). It is hypothesized that circulating BMDCs play a pivotal role in the various body. The unique roles of BMDCs in migrating to various organs have been demonstrated by numerous researchers, including our group. For example, we determined that BMDCs migrating to the brain assemble specifically in the paraventricular nucleus (PVN) during fasting to regulate appetite by secreting brain-derived neurotrophic factors (BDNFs) to maintain homeostasis (Urabe et al., 2013). In contrast, BMDCs migrating to the PVN are significantly decreased, and crosstalk with neuronal cells is impaired in diabetes mellitus, a state of disrupted homeostasis (Katagi et al., 2021; Katagi et al., 2023). Furthermore, migrating BMDCs have been shown to differentiate into interstitial cells of Cajal, which govern gastrointestinal motility, at a steady state but significantly decrease in number in diabetes mellitus (Li et al., 2011). Additionally, the functional role of BMDCs in normal and injured conditions has been reported in other organs, such as the liver, kidneys, and bones (Kojima et al., 2004; Fujimiya et al., 2007; Kasahara et al., 2010; Takemura et al., 2012; Nobuta et al., 2019).

Conversely, BMDCs migrating to the epidermis of the skin have been reported to be rare (Inokuma et al., 2006; Tamai et al., 2011). Compared with the results in other organs, this finding is notable in the skin, although it may be explained by the specificity of the skin, which is located in the most superficial region of the body. This prospective study focuses on the specific phenomena of BMDCs in the skin.

Skin regeneration after deep injuries accompanied by soft tissue defects (hereafter referred to as deep injuries) is a challenge for researchers as well as a hope for patients suffering from deep injuries or ulcers due to trauma, radical dissection due to malignant tumors, or burns. To address this issue, research and development of novel scaffolds have been intensively performed. To date, over 75 skin substitutes have been able to be utilized in the US (Vecin and Kirsner, 2023). However, scaffolds that enable skin regeneration are yet to be developed. We describe general information about scaffolds that is useful for clinicians and, finally, propose strategies to accomplish scaffolds beyond surgical treatments.

## Role of BMDCs in organs at a steady state

To investigate the behavior and function of BMDCs in an organ of interest, bone marrow transplantation (BMT) rodents receiving allogenic BM cells from GFP animals have been frequently used to visualize the dynamics of BMDCs. As controls, the data of BMT animals were compared with those of BMT animals with diseases, injuries, or genetic mutations. However, it is questionable whether BMT animals correspond to *bona fide* controls in skin research. This is because moderate-dose irradiation (∼9 Gy), necessary for the elimination of recipient BM cells, causes skin inflammation due to radiation injury (Okano et al., 2015). Consequently, inflammation induces BMDCs migration, most of which involves immune cells derived from BM. Hence, BMDCs observed in the skin of BMT animals might not reflect those observed under homeostatic conditions. This hypothesis is supported by an experiment involving BMT mice whose heads were protected by lead when radiation was administered to eliminate BMDC in donor mice (Okano et al., 2021). Interestingly, BMDCs were rarely detected in the dermis or epidermis of the head skin, which is distinct from BMT animals without lead. Notably, a lack of valine, an essential amino acid, causes hematopoietic stem cell (HSCs) depletion (Taya et al., 2016). BMT was successfully performed without radiation using a valine-free diet. Thus, the analysis of BMDCs in the skin by using this system might shed a light on the current concept of the role of BMDCs in the skin at a steady state, while the efficacy of BMT is <30%, which is significantly lower compared with conventional BMT model utilizing irradiation, whose efficacy is >80% (Okano et al., 2015; Kang et al., 2020).

Furthermore, considering that keratinocytes are highly differentiated cells, potential GFP-positive keratinocytes have the possibility that their GFP promoter might be inactivated during differentiation process from basal cells to corneocytes in order to express restricted genes for differentiation. To overcome this limitation, Y chromosome staining is used to detect BMDCs, utilizing BMT female animals in which BMDCs from male GFP animals were transplanted (Rendl et al., 2008). However, radiation injury to replace recipient BMDCs with donor BMDCs is unavoidable, even with this method. Therefore, the behavior and function of BMDCs in the skin at a steady state remain unclear.

## BMDCs contribution in response to skin injuries

### Process of wound healing

Wound healing is an extremely complicated and dynamic process in which various cell populations participate. Furthermore, receptors, secreted proteins, or enzymes expressed by these cells are dependent on numerous factors, such as timeline, depth/size of wounds, or types of injury. Brief information on wound healing will be described in this review because detailed information and discussion have been provided in many reviews (Broughton et al., 2006; Yildirimer et al., 2012). Wound healing occurs in three different stages: inflammation, proliferation, and remodeling. Once an injury deeper than the full thickness occurs, vessel rupture leads to bleeding, which forms a fibrin clot after platelet activation and the coagulation cascade (Noris and Galbusera, 2023). Importantly, the fibrin clot functions as a natural scaffold in which immune cells such as neutrophils, monocytes, and fibroblasts, can infiltrate, cope with bacterial invasion, and eliminate numerous dead cells (Broughton et al., 2006). Most monocytes in wounds differentiate into macrophages (Broughton et al., 2006). Notably, M1 macrophages have the capacity to secrete inflammatory cytokines to fight bacteria or pathogens during the inflammatory phase, whereas they are replaced by M2 macrophages, which play a role in tissue repair during the proliferation and remodeling phases (Krzyszczyk et al., 2018). Fibroblasts, myofibroblasts, and vascular cells accumulate to form granulation tissues during the proliferative phase (Broughton et al., 2006). Lymphangiogenesis plays an integral role in wound healing (Park et al., 2011). At the wound edge, keratinocytes are stimulated to proliferate and migrate toward the defect. Finally, wound healing is accomplished through the reorganization of type I collagen fibers from type III collagen fibers and wound contraction during the remodeling phase.

Remarkably, BMDCs contributed to all three processes of wound healing to restore homeostasis. Here, we discuss the role of BMDCs in wound healing.

### Irradiation injuries

Radiation can be ionizing: X- and gamma-rays, which are commonly used for treatments in clinical settings; non-ionizing: ultraviolet light, which is included in sunlight and causes DNA damage in the skin.

There has been an increase in the demand for radiation therapies using ionizing radiation for patients with cancer (Baskar et al., 2012). In this process, exposure of the skin to ionizing radiation is inevitable, resulting in erythema or desquamation as adverse effects at early stages but incurable ulcers or radiation-induced cancer at later stages (Jaschke et al., 2017). Inflammation caused by exposure to ionizing radiation leads to an increase in the number of Langerhans cells in the epidermis, dendritic cells in the dermis in a dose-dependent manner, and BMDCs in the skin (Cummings et al., 2012). Notably, Langerhans cells are replenished by resident Langerhans cells, while other types of dendritic cells are derived from the bone marrow (Merad et al., 2002). Intriguingly, BMDCs in the dermis include myofibroblasts as well as dendritic cells because a substantial population of BMDCs are alpha smooth muscle actin (αSMA)-positive, a myofibroblast marker (Hinz et al., 2007). Conversely, BMDCs which migrate into the epidermis in response to ionizing radiation during the remodeling phase are different from Langerhans cells, despite being positive for Langerin, which is a marker for Langerhans cells. In other words, *Langerin*-positive BMDCs express *Arg-1*, *Retnla*, and *Ym-1*, all of which are M2 macrophage markers, and are never positive in Langerhans cells (Okano et al., 2021). These *Langerin*-positive BMDCs were shown to recover epidermal damage caused by ionizing radiation, as M2 macrophages are crucial for tissue repair in the dermis via the CCL17-CCR4 pathway.

The skin, which is the most superficial organ covering the body, is exposed to sunlight, which involves non-ionizing radiation, partially composed of ultraviolet A (UVA) at 320–420 nm and UVB at 275–320 nm in wavelength. The difference in wavelength leads to varied penetration of the dermis for UVA and of the epidermis for UVB. Both UVs induce radiation injury in the skin because UVA damages the stroma in the dermis by generating free radicals and UVB mutates DNA in keratinocytes located in the epidermis. Consequently, excess sunlight exposure could cause “photoaging” which is characterized by elastic reduction or increasing wrinkle formation (Huang and Chien, 2020). Additionally, photoaging skin could be a source of skin cancers, such as squamous cell carcinoma, basal cell carcinoma, and melanoma. Alternatively, UV radiation disrupts not only the skin microbiome balance but also the skin barrier function to allow microbes to invade the skin, leading to an immune response. In turn, migration to sentinel lymph nodes by immune cells, such as Langerhans cells, dendritic cells, or macrophages, results in their depletion in the skin (Wo et al., 2020). Subsequently, immune cells of bone marrow origin replenish injured skin to maintain homeostasis (Nguyen and Soulika, 2019). Notably, resident Langerhans cells proliferate in response to ionizing radiation, whereas BMDCs differentiate into Langerhans cells in response to non-ionizing radiation. These phenomena strongly indicate the plasticity of BMDCs, to recover homeostasis throughout the body.

In contrast, BM-derived mesenchymal stem cells (BM-MSCs) migrate in the skin from UV-induced injury at the following remodeling phase (Lopez Perez et al., 2019). Notably, the supernatant of the BM-MSCs culture ameliorated moisturization and the depth of wrinkles in the skin caused by UV injury, probably due to enriched exosomes containing a variety of miRNAs, cytokines, or growth factors (Kwon et al., 2016).

Taken together, BMDCs play an outstanding role as “versatile players” in recovering homeostasis from skin injuries.

### Wound healing

Unlike irradiation injuries, most skin injuries are accompanied by skin defects, which are called open wounds. In some cases, other tissues such as subcutaneous tissue, fascia, muscle, or bone are also damaged.

In the inflammatory phase, BM-derived immune cells focus on fighting invasive microorganisms and dead cells (Nguyen and Soulika, 2019). Recently, a novel wound-healing model was proposed for deep wounds. In such injuries, the fascia, not fibrin clots, serves as a scaffold by steering the surrounding tissue (Correa-Gallegos et al., 2019). Specifically, vessels and nerves are relocated in the wound with a steered fascia, and the source of the steering force is myofibroblasts. Thus, a sufficient supply of myofibroblasts in the fascia, the origin of which is substantially from the BM, as well as from local fibroblasts, is required for wound healing of deep injuries (Li, 2009). However, myofibroblast remodeling results in fibrosis, which frequently causes functional and cosmetic problems in clinical practice. In mice, myofibroblasts expressing *Engrailed-1* (*En1*) are shown to be responsible for scar collagens, and so the elimination of *En1*-positive myofibroblasts facilitates scarless wound healing, although their origin remains unknown (Mascharak et al., 2021). Further investigation of the origin of the myofibroblasts responsible for scar formation is necessary to apply the results of this scientific research in a clinical setting. In contrast, the contribution of BMDCs to wound healing after the inflammatory phase has been reported. For instance, skin defects after burn injury of more than 1 cm^2^ were accompanied by accumulation of BM-derived fibroblasts with more than 50% of total fibroblasts, as well as small population of BM-derived keratinocytes in mice (Rea et al., 2009). Alternatively, in a relatively small burn with 3 mm margins BMDCs sporadically observed during the remodeling phase were collagen I- and αSMA-negative, indicating that they were not a myofibroblast lineage (Rea et al., 2009). These findings strongly suggest that skin tissue repair by BMDCs depends on the severity grade. More precisely, non-severe wounds can be sufficiently repaired by local cells, whereas BMDCs contribution is required in severe wounds. This hypothesis is plausible considering that non-severe wounds can be treated conservatively, whereas surgical intervention, such as tissue transfer from other regions, is required for severe wounds in clinical practice. Notably, grafted skin allows BMDCs migration, leading to regeneration by BM-derived keratinocytes and BM-derived hair follicles in the skin graft in mice (Tamai et al., 2011), while skin appendages, such as hair follicles, are never regenerated in grafted skin in humans.

Overall, the plasticity of BMDCs is maximized in the process of wound healing, which appears to depend on the external environments in addition to the internal environments.

## Clinical use of scaffolds for skin injuries

As discussed in the previous section, synthesis of extracellular matrix (ECM) by myofibroblasts during the remodeling phase in a wound is an inherent healing mechanism in the body (Klingberg et al., 2013). However, impaired wound healing frequently occurs in patients in a clinical setting because of their background, such as diabetes or autoimmune diseases, prolonged inflammation or infection, or huge skin defects in patients with burns, which are called intractable ulcers. Alternatively, even if deep skin injuries heal, ECM deposition in the tissue never recovers to normal tissue but scars. Furthermore, excess ECM accumulation can cause either keloid or hypertrophic scarring, which causes both functional and cosmetic problems in patients.

Hence, surgical treatments utilizing flap(s) or skin grafts are not only applied to patients with impaired wound healing, that is, intractable ulcers, but scar revision is also performed in patients with keloid/hypertrophic scars. However, current goal is to either recover defects with autografts or improve scar appearance without regenerating the lost normal tissue. In this regard, tissue regeneration has not been accomplished using surgical treatment, suggesting the need for different approaches. Furthermore, methods that are less invasive than surgery are beneficial to patients.

With this background, the application of scaffolds to wounds is a promising option to shed some light on these problems; therefore, researchers have intensively studied them over the decades. Natural biomaterials, synthetic materials, and natural-synthetic hybrid materials are used in scaffolds, although most of the last two have been studied in laboratory animals (Klingberg et al., 2013). Alternatively, from a theoretical viewpoint, scaffolds alone, scaffolds in which cells are seeded, or scaffolds containing growth factors can be constructed, although the first two types of scaffolds have been utilized in clinical settings. In this review, an outline of scaffolds used in clinical practice is described, and detailed information on scaffolds *per se* has been discussed elsewhere (Qin et al., 2022; Vecin and Kirsner, 2023).

### Scaffolds without seeding cells or growth factors

Scaffolds made from bovine or porcine collagen cross-linking have been the first choice for impaired wound healing because animal collagen matrices have been reported to be useful for the formation of the neodermis in wounds (Burke et al., 1981). Allograft acellular dermal matrices are currently available (Yildirimer et al., 2012; Vecin and Kirsner, 2023). The application of animal collagen matrices allows myofibroblasts to migrate and synthesize ECM during deep injuries. This status is called the neodermis, although the neodermis is different from native granulation tissue with respect to thickness and pliability (Chang et al., 2019). A split-thickness graft is necessary a few weeks after the application of animal collagen matrices, while the cosmetic appearance is superior to a split-thickness graft alone on the defects (Chang et al., 2019; Petrie et al., 2022). Patients with extensive burns would be good candidates for management using scaffolds, considering the limited donor skin for autografts. However, treatments with scaffolds have not been frequently used for patients with burns, probably because the high cost of these scaffolds limits their use for extensive wounds or emergent use.

Nevertheless, scaffolds without seeding cells or growth factors are still an attractive tool, considering that they can be more easily and quickly applied to patients than cell-incorporated scaffolds. For example, we recently demonstrated the unexpected result that the use of a gelatin sponge as a scaffold led to tissue regeneration in deep injuries with periosteal defects in the calvariae of rats (Shirai et al., 2022). Surprisingly, gelatin sponges allowed periosteum regeneration as well as skin appendages such as hair follicles or peripheral nerves, whereas the defects were encrusted in wounds without gelatin sponges. We unraveled the key mechanism by which microenvironments provided by gelatin sponges switched the differentiation fate of BMDCs from myofibroblasts to endothelial cells, pericytes, and vascular smooth cells that would have differentiated into fibroblasts in wounds without gelatin sponges, although further studies are needed. This vasculogenesis was followed by tissue regeneration as well as the appendages regeneration in the skin. The study on the precise factors in gelatin sponges that allowed such skin regeneration has been ongoing in our laboratory. Since gelatin sponges have been used as a hemostatic material in a clinical practice, repositioning of this material will be an easy and smooth process of clinical use. Conversely, the disadvantage is that the pore sizes of gelatin sponges cannot be regulated as far as we reposition them.

### Cell-incorporated scaffolds

In the late 1990s, the FDA approved cell-incorporated scaffolds for intractable ulcers, expecting recovery of the natural function and structural characteristics of human skin (Eaglstein and Falanga, 1998; Waymack et al., 2000; Curran and Plosker, 2002). Since then, similar types of scaffolds have been developed in which allogeneic cultured fibroblasts and/or keratinocytes are seeded in three dimensional dermal substitutes (Eaglstein and Falanga, 1998; Waymack et al., 2000; Curran and Plosker, 2002; Bay et al., 2021; Vecin and Kirsner, 2023). Notably, cell-seeded scaffolds could be rejected owing to the immune response. The reason why graft-versus-host disease seldom occurs in the case of these scaffold applications is probably due to the depletion of antigen-presenting cells from donors, such as Langerhans cells (Eaglstein and Falanga, 1998). Despite this, the lack of immune rejection against scaffolds in most patients is surprising, possibly because seeded cells do not survive in implants, which is supported by the absence of keratinocytes or fibroblasts responses in matrices (Curran and Plosker, 2002; Schurr et al., 2012). However, these studies are inconsistent with other studies (Eaglstein and Falanga, 1998). The fact that split-thickness grafting could be necessary simultaneously or after the application of these scaffolds suggests that the effect of seeded cells is temporary (Waymack et al., 2000; Dixit et al., 2017), although the timing of cell death remains unclear.

Human placenta-derived skin substitutes can also be used by clinicians. The first clinical use of placental tissue was skin grafting in 1910, which showed better results than xenografts or cadaveric skin grafts (Silini et al., 2015). Since the 1950s, placenta-derived scaffolds, including the amniotic sac, trophoblast layers, and their combinations, have been used to treat skin injuries. Thus far, numerous placenta-derived biomaterials are available for wound healing, depending on the source (amnion, chorion, trophoblast layers, umbilical cord, or their combination), preservation methods (dehydration or cryopreservation), or cell status (containing viable cells or decellularization) (Protzman et al., 2023). The advantages of placenta-derived scaffolds over human fibroblast-derived scaffolds involve a lower cost [$3846 for placenta-derived scaffolds or $7968 for human fibroblast-derived scaffolds per patient (Ananian et al., 2018)] and reliance on their use for treatments over long periods. Additionally, immune-compatibility in placenta-derived scaffolds should be noted, as Azizian et al. (2018) showed that the expression of MHCI and HLA-DR in amniotic epithelial cells, those of which are receptors recognized by recipient T cells, remained extremely low in 3D scaffolds. Placenta-derived cells exhibit anti-inflammatory effects. For example, placenta-derived cells suppress the activation and proliferation of T cells, reduce Th1 inflammatory cytokines, and induce T-cell regulation (Silini et al., 2015). Indeed, clinical trials performed for chronic diabetic foot ulcers in 2017 showed that wounds healed significantly in cases treated with placenta-derived scaffolds compared to those treated with human fibroblast-derived scaffolds (Ananian et al., 2018).

However, the fact that the source of placenta-derived scaffolds is the human or animal organ associated with placentae not only limits their extensive use but also raises ethical concerns. Moreover, unknown viruses that cannot be detected by viral tests may be included in placenta-derived scaffolds. Some cancers can be caused by viruses, such cervical cancer caused by human papilloma viruses, therefore, the risk of undetectable viruses is crucial in utilizing placenta-derived scaffolds.

## Disadvantages of scaffolds

Clinicians have several choices for scaffolds in treating wounds, but there are still critical problems with scaffolds that need to be addressed to surpass surgical treatments.

First, the scaffolds cannot be used to treat infected wounds. Wounds dressed with scaffolds provide an adequate environment for bacteria as well as wounds *per se*. Once a bacterial infection cannot be regulated, the removal of scaffolds is urgently necessary to avoid deeper and more severe wounds because of the accelerated population of bacteria. Consequently, patients suffer further from intensive care as well as a prolonged period prior to wound healing. Recently, scaffolds coated with metal ion nanoparticles (NP), especially silver (Ag) NPs, have been used to treat bacterial infections. The mechanism of action of AgNPs against bacteria has not been fully determined, although they can work in various ways, such as rupturing cell membranes and inactivating and/or destroying enzymes, DNA, or proteins in bacterial cells (Qing et al., 2018). Nevertheless, virulent bacteria can overwhelm the action of AgNPs in wounds. In such cases, even scaffolds containing AgNPs become a suitable environment for bacterial growth. When virulent bacteria form biofilms, aggregated bacteria are surrounded by a self-produced matrix attached to a wound, and AgNPs are unable to approach the biofilm. Furthermore, a distinct disadvantage of AgNP-coated scaffold development is the difficulty in regulating proper Ag diffusion in wounds, an excess of which can be cytotoxic to human cells. This might be one of the reasons why there are few AgNP-coated scaffolds for skin injuries, although more than 10 years have passed since Yildirimer et al. (2012) concluded that ideal antibacterial scaffolds coated with AgNPs were not available as of 2012 in their review. Therefore, the development of novel scaffolds that are impervious to bacteria-induced infectious wounds is crucial to surpass surgical treatment. This challenge should be urgently addressed considering that surgical debridement, the first option currently available for infectious wounds, involves an *en-bloc* excision of infectious tissue. As the lost tissue should never be regenerated after debridement, subsequent surgery for reconstruction using either a skin graft or subcutaneous flap is imperfect. For instance, skin grafting on lost subcutaneous tissue lacks elasticity and sensory properties, whereas reconstruction using a flap is accompanied by donor sacrifice. Thus, novel scaffolds that can be applied to infectious wounds could lead to breakthroughs in the treatment of deep wounds.

Since the discovery of penicillin in 1928, antibiotics have become powerful tools for treating infectious diseases, including infectious wounds. However, the indiscriminate administration of various antibiotics worldwide has increased the prevalence of antibiotic-resistant bacteria. Hence, the application of scaffolds containing specific antibiotics to protect wounds from bacterial infection could cause the immediate emergence of multidrug-resistant bacteria, which would be the reason why such scaffolds, including antibiotics, are not available. Moreover, antibiotics, similar to AgNPs, cannot penetrate biofilms.

Second, the goal of using scaffolds today is not to regenerate the skin but to accelerate wound closure and/or to obtain a superior cosmetic result compared to the one without scaffolds. In other words, the scaffolds set in the wounds were replaced by an accumulation of ECM with no skin appendages. Hence, the healed region is vulnerable to external stimuli that can cause recurrence. Otherwise, cosmetic problems could lead patients to undergo surgical scar revisions, resulting in an additional period and economic costs. Various efforts have been made to overcome these advantages, some of which are within the clinical pipeline. For example, the application of siRNA against connective tissue growth factor, which is overexpressed in hypertrophic scars/keloids, was successful in preventing hypertrophic scars/keloids (Kang et al., 2020). Another clinical pipeline utilizing fibromodulin (FMOD) peptide sequences against hypertrophic scars/keloids is ongoing because of their positive effects on endothelial cell migration and myofibroblast differentiation to accelerate wound healing (Jiang et al., 2018). Connexin43 mimetic peptide is also noted because its use prevents the formation of hypertrophic scars/keloids by competing with naïve connexin 43, a component of hypertrophic scars/keloids (Montgomery et al., 2018). Although these wound therapies have not focused on scaffolds, it may be intriguing to utilize scaffolds as an option for drug delivery systems, considering that these molecules or peptides are already in the clinical pipeline.

Altogether, attempts to combat these disadvantages are required for scaffolds to be used as a priority in wound treatment.

## Development of scaffolds which make tissue regeneration possible in deep injuries

**FIGURE 1:**
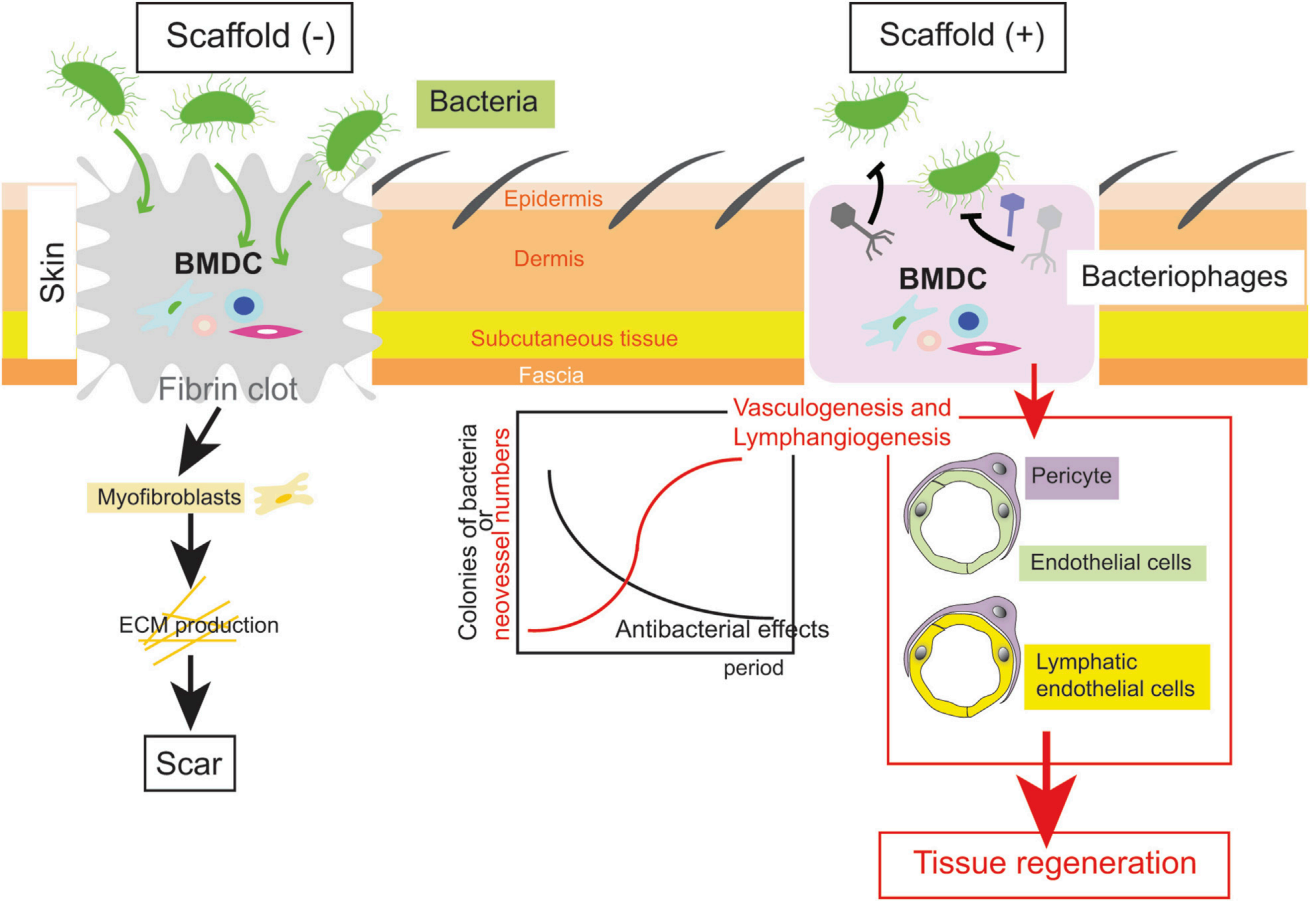
An example of a scaffold to regenerate tissue after a deep injury. In an ideal scaffold, while antibacterial effects by a bacteriophage cocktail are efficient, vasculogenesis and lymphangiogenesis is completed (the right scheme). Thereafter, epidermis/dermis including skin appendages are regenerated. Conversely, ECM deposition by myofibroblasts results in scar during conventional wound healing (the left scheme).

In order to provide scaffolds that allow tissue regeneration even in deep injuries in a clinical setting, we propose an important strategy, namely, maximal utilization of the inner homeostasis of the body (Figure 1).

First, it is necessary to develop biodegradable scaffolds that can be applied to infectious wounds. Unlike metal ions or antibiotics, it would be a great innovation if a substance that kills bacteria protected by biofilms could be combined in scaffolds. One of these options might be the use of bacteriophages, which have been used in clinical pipelines to treat infectious wounds (Freedman et al., 2023). Bacteriophages, whose name indicates “bacteria-eaters” in Greek, are estimated as more than 10^31^ in number, the biggest population among all organisms on the Earth (Keen, 2015). Therefore, bacteriophages inevitably exist against each bacterial species, and notably, the actions of bacteriophages are limited to their target bacteria, unlike antibiotics, which indiscriminately affect numerous bacteria. Furthermore, bacteriophages can penetrate biofilms without affecting eukaryotic cells. This specificity of bacteriophages could prevent the emergence of multidrug-resistant bacteria as a result of the abuse of antibiotics when bacteriophage therapy is available. Indeed, the disadvantage that bacteria are rapidly resistant to one kind of bacteriophage can be overcome by combining several kinds of bacteriophages (phage cocktail) (Luong et al., 2020). Since phage cocktails cured a patient severely infected with multidrug-resistant *Acinetobacter baumannii* in the US in 2017 (Schooley et al., 2017), research in the field of bacteriophages has been growing in the hope of novel therapies for multidrug-resistant infectious diseases. Thus, scaffolds containing a phage cocktail could be inventable, allowing their application to infectious deep wounds with the aim of tissue regeneration.

Second, the development of appropriate scaffolds which enable BMDCs to migrate into wounds is necessary. In this regard, BMDCs are frequently confused with MSCs, which are also used as a seeded cell resource within scaffolds. As discussed in the previous sections, BMDCs include not only MSCs but EPCs which differentiate in endothelial cells necessary for vasculogenesis and HSCs which differentiate into cells other than blood cells, osteoblasts and osteoclasts (Kloc et al., 2022). In addition, differentiation of BMDCs can lead to lymphangiogenesis as well as vasculogenesis (Biswas et al., 2023). Lymphangiogenesis has been shown to be efficient to the impair wound healing in the skin of mice (Güç et al., 2017). Overall, targeting BMDCs rather than MSCs appears to be efficient in the development of novel scaffolds.

Finally, it is important to focus on scaffold-related factors, such as biodegradability, viscoelasticity, or topological structures that can affect the fate of BMDCs migrating into scaffolds. For example, the viscoelasticity of scaffolds regulates MSC chondrogenesis, because matrix viscoelasticity modulates cell morphology and actin organization (Huang et al., 2019). This finding indicates that viscoelasticity determines the migration speed and the status of pseudopodia (Cantini et al., 2020). These research fields investigating the relationship between cell fate determination and rheology will shed the light on the development of novel scaffolds in which BMDCs migrate efficiently.

In conclusion, scaffolds that disinfect bacteria during vasculogenesis and lymphangiogenesis by regulating the fate of BMDCs will enable tissue regeneration even in infectious deep injuries.

## Summary

Owing to the specificity of the skin, which is the outermost part of the body, repair and recovery from injuries can occur constantly to maintain homeostasis. Although almost all local skin components participate in wound healing, BMDCs, which migrate dynamically and exhibit plasticity depending on the situation, should also be emphasized as another player. The application of scaffolds in wounds, a nonsurgical therapy, is superior to surgery with respect to noninvasive treatments. However, further progress is required to surpass surgery. The regulation of BMDCs migration in wounds could shed light on overcoming the disadvantages of scaffolds.

## Data Availability

The original contributions presented in the study are included in the article, further inquiries can be directed to the corresponding author.
